# An Outlier Detection Method Based on Mahalanobis Distance for Source Localization

**DOI:** 10.3390/s18072186

**Published:** 2018-07-07

**Authors:** Qingli Yan, Jianfeng Chen, Lieven De Strycker

**Affiliations:** 1 School of Marine Science and Technology, Northwestern Polytechnical University, Xi’an 710072, China; chenjf@nwpu.edu.cn; 2 KU Leuven, ESAT-DRAMCO, Ghent Technology Campus, 9000 Ghent, Belgium; lieven.destrycker@kuleuven.be

**Keywords:** angle of arrival, source localization, outlier detection, Mahalanobis distance, unreliable nodes

## Abstract

This paper addresses the problem of localization accuracy degradation caused by outliers of the angle of arrival (AOA). The problem of outlier detection of the AOA is converted into the detection of the estimated source position sets, which are obtained by the proposed division and greedy replacement method. The Mahalanobis distance based on robust mean and covariance matrix estimation method is then introduced to identify the outliers from the position sets. Finally, the weighted least squares method based on the reliable probabilities and distances is proposed for source localization. The simulation and experimental results show that the proposed method outperforms representative methods when unreliable AOAs are present.

## 1. Introduction

The source localization techniques based on the angle of arrival (AOA) estimate the target position using a set of estimated bearings. Various methods have been proposed to solve the localization problem [1,2,3], among which the closed-form method of the pseudolinear estimator (PLE) was proposed under the assumption that AOA errors are small [4]. Although the PLE method is easy to implement and is efficient to compute, it is sensitive to outliers (AOAs with large errors), and such outliers (also referred to as unreliable AOAs) may exist in many practical applications of distributed node networks, which typically consist of a large number of small, low-cost sensor nodes. When nodes are deployed in harsh and unattended environments, animal attack or other forms of interference may occur. Moreover, low-cost nodes have limited amounts of power, computational, and memory capacity, and these limitations may also cause outliers. Other factors, such as node failures, data loss, and non-line of sight (NLOS) propagation [5,6], can lead to unreliable measurements. As a result, the estimated AOAs at each node will deviate significantly from the true values. Such outliers have been found to be detrimental to the PLE [7,8]. Thus, it is important to identify these erroneous data to improve the localization performance or perform a repair of the data.

To reduce the error induced by outliers in node networks, several hybrid localization methods have been proposed by combining the AOA with the time difference of arrival (TDOA) and received signal strength (RSS) to identify and mitigate the NLOS error [9]. The expectation maximization (EM) method is introduced to identify unreliable AOAs caused by NLOS [10]. The intersection points (IPs)-based method [11] calculates the source position by taking the centroid of the set of intersections obtained by pairs of bearing lines; however, this method cannot significantly improve the localization performance, even eliminating the IPs obtained by two bearing lines close to parallel. The proposed unreliable AOA detection method in [7] can improve the localization accuracy; however, many threshold parameters need to be set. The steered-response power phase transform (SRP-PHAT) [12] source localization approaches have demonstrated robustness when operating in reverberant and noisy environments. Regardless, these methods require a considerably higher amount of information to be transmitted to the central processing node and cannot be applicable to large-range localization scenarios (e.g., hundreds or thousands of meters). In this work, every node is equipped with a microphone array to estimate the AOA and then transmits the estimated AOA to the central node. This method does not require time synchronization in different nodes. Note that AOA estimation methods under an environment with complex environmental noise are outside the scope of this paper; interested readers are referred to [13,14,15]. These robust AOA estimation methods are proposed under the assumption that only small portions of snapshots are contaminated; they can perform well for continuous source signals or impulsive interference noise, which only have influence on limited snapshots. However, with other causes that can last a period of time, such as sensor failures and non-line of sight (NLOS) propagation, all snapshots for one node are unreliable; thus, outliers that may deteriorate the localization performance are still present even when these methods are applied to estimate AOAs under complex noise. Therefore, the outlier detection for the AOA is still necessary to improve the localization accuracy.

Here, we propose a robust localization method when outliers are present. A large number of positions can be obtained by different node combinations. The maximum number of estimated positions is *N* × (*N* − 1)/2 for an *N*-node network. However, the estimated positions are sensitive to the bearing lines and their differences. Deleting the outliers from all intersections alone cannot significantly improve the localization performance [11]. To increase the estimated position reliability and improve the detection accuracy, we propose the division and greedy replacement (DIG) method to obtain different estimated position sets by changing one node at one time. The robust estimation method of the mean and covariance matrix for estimated position sets is then addressed to provide the information for outlier detection. The Mahalanobis distance (MD) [16] is finally proposed to identify the outliers from the estimation position sets. Finally, the weighted least squares (WLS) method based on detected reliable probabilities and distances is used to estimate the source position. The proposed method is easy to implement and can be easily extended to a three-dimensional (3D) source localization method. The main contributions of this paper can be summarized as follows:

- The division and greedy replacement (DIG) method is developed to estimate the target positions.
- The Mahalanobis distance based on robust estimation of mean and covariance matrix is proposed to detect the outliers from estimated source positions.
- An improved WLS localization method based on reliable probabilities and distances is introduced.
- Outdoor experiments are conducted to verify the proposed method.

The remainder of this paper is organized as follows. Section 2 describes the AOA localization method and addresses the existing problem. The unreliable node detection method is proposed in Section 3. Simulations and experimental results are presented in Section 4 and Section 5, respectively. Finally, our work is summarized in Section 6.

## 2. AOA-Based Localization Method and Problem Statement

### 2.1. The Pseudolinear Estimator (PLE)

**Figure 1:**
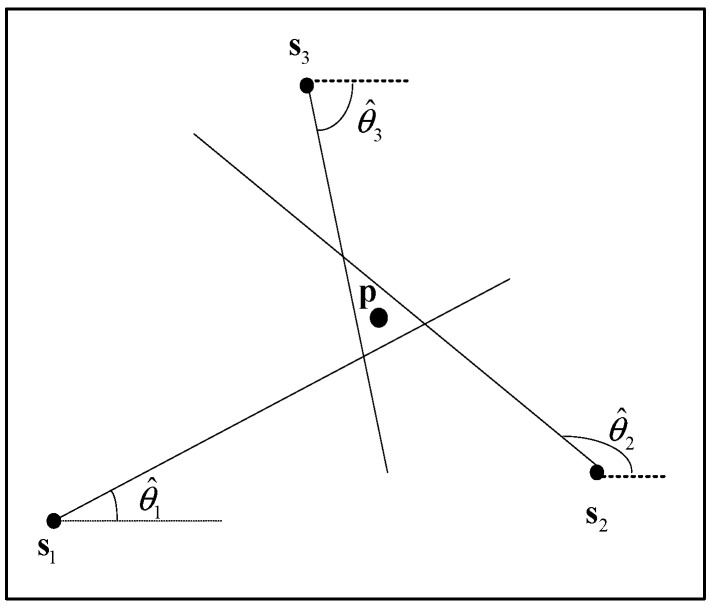
Illustration of angle of arrival (AOA) localization with three nodes deployed in the test area.

We consider *N* nodes equipped with a microphone array for each one, with known positions $s_{k}={\left[x_{k},y_{k}\right]}^{T}$ (*k* = 1, 2, …, *N*), are deployed in an area of interest to estimate the location of a single source $p={\left[x,y\right]}^{T}$ as shown in Figure 1. Under the Gaussian background noise assumption, the estimated angle ${\hat{\theta }}_{k}$ of *k*-th node can be given by the following:

(1) $${\hat{\theta }}_{k}=\theta_{k}+\eta_{k},$$

where

(2) $$\theta_{k}=\arctan(\frac{y-y_{k}}{x-x_{k}}),$$

and $\eta_{k}$ is the zero mean Gaussian noise with variance $\sigma_{i}^{2}$.

The set of measurements from *N* nodes can be written as follows:

(3) $$\hat{\theta }=\theta +\eta ,$$

where $\hat{\theta }={\left[\begin{array}{cccc} {\hat{\theta }}_{1} & {\hat{\theta }}_{2} & \ldots & {\hat{\theta }}_{N} \end{array}\right]}^{T}$, $\theta ={\left[\begin{array}{cccc} \theta_{1} & \theta_{2} & \ldots & \theta_{N} \end{array}\right]}^{T}$, and $\eta ={\left[\begin{array}{cccc} \eta_{1} & \eta_{2} & \ldots & \eta_{N} \end{array}\right]}^{T}$. Thus, the pseudolinear estimator (PLE), also known as the orthogonal vectors (OV) estimator, can be used to estimate the source position and is given by the following [4]:

(4) Ap=B+e,

where the estimated source position is as follows:

(5) $$\hat{p}={(A^{T}A)}^{-1}A^{T}B,$$

where the *k*-th row of matrix **A** and **B** is $A(k,:)=[\begin{array}{cc} \sin{\hat{\theta }}_{k} & \cos{\hat{\theta }}_{k} \end{array}]$, $B(k,:)=x_{k}\sin{\hat{\theta }}_{k}-y_{k}\cos{\hat{\theta }}_{k}$, *k* = 1, 2, …, *N*, and

(6) $$e={\left[\begin{array}{cccc} r_{1}\sin\eta_{1} & r_{2}\sin\eta_{2} & \ldots & r_{N}\sin\eta_{N} \end{array}\right]}^{T},$$

where $r_{k}$ is the distance between the source and node $s_{k}$.

### 2.2. Problem Formulation

The PLE is easy to implement, even for large-scale data. However, the PLE is sensitive to unreliable measurements (i.e., outliers). In this section, we use theoretical analysis and simulation results to illustrate this problem.

If the measurement error is sufficiently small, then we have $\sin\eta_{k}\approx \eta_{k}$. Thus, the approximation of the residuals of Equation (6) can be expressed as $e_{k}\approx r_{k}\eta_{k}$. The estimated error of the source position can be expressed as follows [8]:

(7) $$\begin{array}{cc} \Delta p & =\hat{p}-p\\ & ={\left(A^{T}A\right)}^{-1}A^{T}B-{\left(A^{T}A\right)}^{-1}A^{T}Ap\\ & ={\left(A^{T}A\right)}^{-1}A^{T}(-e) \end{array}.$$

The covariance matrix of Equation (7) can be obtained by the following:

(8) $$\mathrm{cov}(\Delta p)=E(\Delta p\Delta p^{T}).$$

Thus, the mean-square error (MSE) is given by the following:

(9) MSE=tr[cov(Δp)].

Submitting Equations (6) and (7) into Equation (10), we have the following:

(10) $$\begin{array}{cc} MSE & =\frac{1}{\sum_{i,j\in s}\sin^{2}(\theta_{i}-\theta_{j})}\\ & \cdot \sum_{i\in s}\left\{\sigma_{e_{i}}^{2}[{\left(f_{11}\sin\theta_{i}-f_{12}\cos\theta_{i}\right)}^{2}+{\left(f_{21}\sin\theta_{i}-f_{22}\cos\theta_{i}\right)}^{2}]\right\} \end{array},$$

where *S* is defined as all the combinations if {i,j} with j>i. $f_{11}=\sum_{i\in s}\sin^{2}\theta_{i}$, $f_{22}=\sum_{i\in s}\cos^{2}\theta_{i}$ and $f_{21}=f_{12}=\sum_{i\in s}\cos\theta_{i}\sin\theta_{i}$ and $\sigma_{e_{i}}^{2}=E(ee^{T})$.

We can see from Equation (10) that the MSE is affected by the relative geometry between the source and the nodes, the number of nodes, and the AOA measurement errors. To illustrate the effect of outliers, we conducted several simulations to analyze the characteristics of the localization error for different source positions. The source is assumed to be located at the gridded points, ranging from −10 m to 10 m in a 20 × 20 m^2^ grid with a resolution of 0.5 m. Four nodes—$s_{1},s_{2},s_{3}$, and $s_{4}$—are randomly deployed in the test area, as shown in Figure 1. The root-mean-square error (RMSE) of the PLE of 500 trials for every target position is used as the performance metrics.

**Figure 2:**
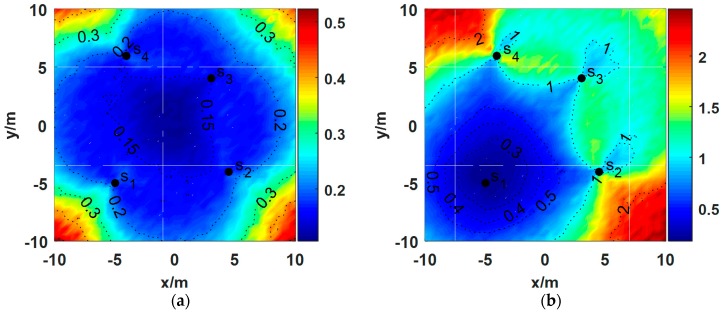
Root-mean-square error (RMSE) for different source positions when four nodes are deployed in a 20×20 m^2^ test area: (**a**) $\sigma_{1}=\sigma_{2}=\sigma_{3}=\sigma_{4}=1{}^{\circ}$; (**b**) $\sigma_{1}=10{}^{\circ}$, $\sigma_{2}=\sigma_{3}=\sigma_{4}=1{}^{\circ}$. The black dots denote the nodes, and the numbers on the dotted line contours are the values of the RMSEs.

The RMSEs for different source positions are shown in Figure 2a when $\sigma_{1}=\sigma_{2}=\sigma_{3}=\sigma_{4}{=1}^{{}^{\circ}}$. It is clear that the localization errors are relatively lower when the source is surrounded by the nodes compared to the outside source. The conclusion follows the analysis based on the Cramer–Rao lower bound (CRLB) in [17].

Assume that the unreliable node $s_{1}$ is subject to a large noise with zero means $\sigma_{1}=10{}^{\circ}$ and $\sigma_{2}=\sigma_{3}=\sigma_{4}=1{}^{\circ}$. The resulting RMSEs are plotted in Figure 2b. When the source is close to the unreliable node, the localization accuracy is not significantly deteriorated. However, the RMSEs are significantly increased when the source is far from the unreliable nodes. From Equation (10), we can see that for the same AOA estimation error $\sigma_{i}$, the MSE is mainly influenced by the distance $r_{k}$ between the source and the node $s_{k}$.

To demonstrate the importance of detecting unreliable nodes, the RMSEs of the estimated positions obtained from the four nodes with one being unreliable are compared with the RMSEs when only three reliable observations are used for the source located at $p={\left[3,0\right]}^{T}$ m. As shown in Table 1, the localization errors obtained using only three reliable nodes are significantly lower than those obtained with four nodes, one of which is unreliable. Therefore, it is necessary to detect the unreliable nodes and then remove them to improve the localization accuracy.

## 3. The DIG_MD Method

**Figure 3:**
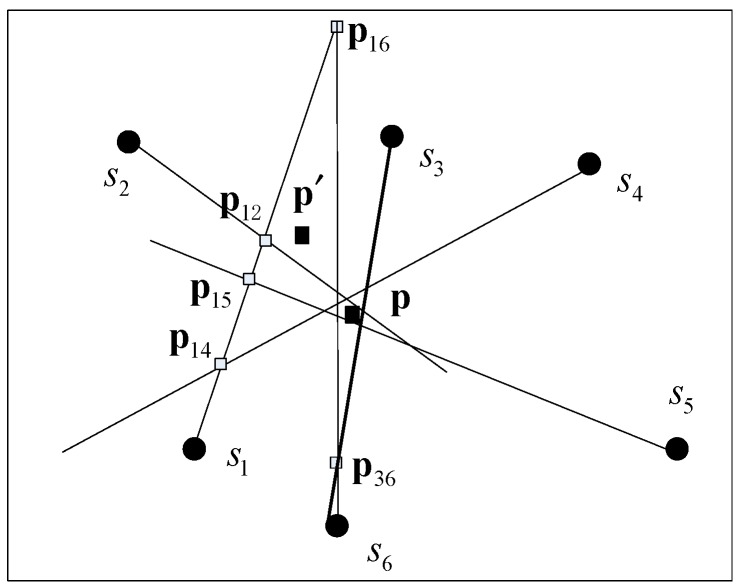
Illustration of the intersection points (IPs) distribution when one bearing is unreliable, where the rectangle denotes the IP and $p_{ij}$ is the intersection point obtained by **s***_i_* and **s***_j_*.

We know that at least two nonparallel bearing lines are required to estimate an IP, and the maximum number of IPs for *N* nodes is N(N−1)/2. Regardless of the parallel cases of two bearing lines, all IPs are expected to be close to each other and surround the true source position when they are only subjected to low-level environment noise. In contrast, the bearings corrupted by large noise will lead the IPs to be far from the source position. As shown in Figure 3, the IPs obtained from $s_{1}$ are obviously far from the other IPs. Therefore, we can identify the unreliable AOAs by detecting outliers from the estimated target positions. However, there are too many intersections to calculate for large-scale node networks if only two bearings are used. Moreover, the IPs are also easily affected by the errors of either one and by the angular distance [11]. For example, it is easy to cause a false alarm if $s_{6}$ is determined to be unreliable when $p_{16}$ and $p_{36}$ are detected as outliers. To solve these problems, the division and greedy replacement (DIG) method is proposed here to improve the stability of the estimated positions. The two-dimensional (2D) outlier detection method is then used to find the unreliable bearings. Finally, the WLS based on detected reliable probabilities and distances from initial position to nodes is used to perform the localization. The procedure is given as follows:

### 3.1. The Division and Greedy Replacement (DIG) Method

In order to detect outliers from the AOAs, based on estimated source positions, a set of position estimations are needed, which should be calculated by a fixed number of nodes only with one independent variable. Thus, every estimated position corresponds to the unique different node. In this paper, we propose to divide all nodes into two sets, and the greedy replacement is then used to obtain different combinations of a fixed number of nodes with one difference.

(1) Division: In this section, the two separated set are defined as the reference node set ($\Omega_{ref}$) and the replacement node set ($\Omega_{rep}$), with sizes *m* and *N* − *m*, respectively. Here, to provide an easier explanation, we assume that the reference nodes are indexed from 1 to *m*. Thus, $\Omega_{ref}$ and $\Omega_{rep}$ can be denoted as $\Omega_{ref}=\left\{s_{1},s_{2},\ldots ,s_{m}\right\}$ (m≥3) and $\Omega_{rep}=\left\{s_{m+1},s_{m+2},\ldots ,s_{N}\right\}$, respectively. Algorithm 1 presents the selection method of reference nodes:

**Algorithm 1.** Selection method of reference nodes.(1)Estimate the initial source position $p^{\prime }$ by the PLE based on all measurements;(2)Calculate the distances from $p^{\prime }$ to all nodes;(3)Select *m* nodes that have short distances and can form a convex polygon with the target inside.

The performance analysis in [17] shows that the nodes that are close to the target are dominant in the localization results and that the localization error for the target inside a convex polygon composed of multiple nodes is smaller than that of an outside one. So, we propose to use the nodes that can comprise a convex polygon with the target inside and have short distances to the source as reference nodes, as shown in step 3; thus, no fewer than three nodes should be selected as the reference nodes. As the true position is unknown, an initial obtained from all the measurements can be used to evaluate the distances stated as step 1 and step 2. As shown in Figure 3, $p^{\prime }$ is the initial position calculated by six measurements; $s_{1},s_{2}$, and $s_{3}$ are closest to $p^{\prime }$, and $p^{\prime }$ is inside the convex polygon formed by the three nodes. Thus, $s_{1},s_{2}$, and $s_{3}$ are selected as reference nodes (i.e., $\Omega_{ref}=\left\{s_{1},s_{2},s_{3}\right\}$ when *m* = 3). The detail of the division method can be summarized as follows.

To identify the unreliable bearings by detecting outliers from a set of estimated positions, we obtain the positions by changing only one node at a time. As noted above, the localization error is sensitive to the bearing error of the nodes relatively far from the source. Thus, we design the greedy replacement method by using each node in $\Omega_{rep}$ to replace one of those in $\Omega_{ref}$. The procedure is given by Algorithm 2. Every node in $\Omega_{ref}$ is replaced by (*N* − *m*) nodes from $\Omega_{rep}$. Next, *m* sets, including (*N* − *m*) positions in each set, can be obtained, and every point is calculated by *m* nodes with (*m* − 1) same nodes from $\Omega_{ref}$. For this method, the position sets can be calculated with cost $Ο(-m^{2}+mN)$. In contrast, the cost is Ο[N(N−1)/2] if all IPs are estimated. In general, the DIG method is computationally simpler than the IP method.

**Algorithm 2.** Greedy Replacement Method. For *k* = 1:m For *j* = m + 1:*N* $\Omega =\Omega_{ref}\cup \{s_{j}\}-\{s_{k}\}$    $p_{k,j}={(A{\left(\Omega \right)}^{T}A(\Omega ))}^{-1}A{\left(\Omega \right)}^{T}B(\Omega )$    $P_{k}(j,:)=p_{k,j}^{T}$  end end

In this paper, X∪Y and X−Y denote the union and difference of sets *X* and *Y*, respectively; A(Ω) represents the matrix **A** in Equation (4) calculated based on the nodes from set Ω; and $P_{k}(j,:)$ is the *j*-th row of $P_{k}$. Each element $p_{k,j}$ of $P_{k}$ is the estimated position using $s_{j},j\in \{m+1,\ldots ,N\}$ to replace $s_{k},k\in \{1,\ldots ,m\}$.

### 3.2. Outlier Detection Method for Estimated Target Position Sets

All the position elements in $P_{k}={\left[p_{k,m+1},p_{k,m+2},\ldots ,p_{k,n}\right]}^{T}$, k=1,2,…,m should be close to each other under the assumption that all the nodes are reliable. The outlier positions should be obtained from the unreliable nodes. For the 2D source localization problem, the elements in $P_{k}$ are identically distributed 2D random vectors with mean $\mu_{k}$ and a positive-definite covariance matrix $\Sigma_{k}$. To identify the unreliable nodes in set $\Omega_{rep}$, the square of the Mahalanobis distance (MD) [18,19,20], which can be formulated as in Equation (11), is proposed to detect outliers from the position matrix in $P_{k}$ as follows:

(11) $$d_{k,j}^{2}(\mu_{k},\Sigma_{k})={\left(p_{k,j}-\mu_{k}\right)}^{T}\Sigma_{k}^{-1}(p_{k,j}-\mu_{k})$$

In the field of data statistics, MD is typically used to characterize how far a particular datum is from the center. A point with a distance greater than a predetermined threshold is assumed to be an outlier. The outlier detection problem in this work is a 2D data detection problem. Therefore, the robust estimated method of $\Sigma_{k}$ and $\mu_{k}$ is important for robust outlier detection. The outlier detection method for one position set $P_{k}$ is given by Algorithm 3.

To better explain Algorithm 3, let us recall the Gnanadesikan Kettenring (GK) estimator first [18], which provides a reasonable relationship between variance and covariance. Assume that V is the covariance matrix of l-dimensional random vector **x** and σ(·) represents the standard deviation; thus, we have

(12) $$\sigma {\left(c^{T}x\right)}^{2}=c^{T}Vc$$

for all $c\in R^{L}$. The GK estimator can be formulated as the following:

(13) $$\mathrm{cov}(x,y)=\frac{1}{4}\left(\sigma {\left(x+y\right)}^{2}-\sigma {\left(x-y\right)}^{2}\right),$$

where **x** and **y** are a pair of random vectors.

**Algorithm**
**3.** Outlier detection method from $P_{k}$.Step 1.Let $D=\mathrm{diag}(\sigma (P_{k}(:,1)),\sigma (P_{k}(:,2)))$, and $M_{k}=P_{k}D^{-1}$.Step 2.Compute the correlation matrix Ψ, $\Psi_{11}=\Psi_{22}=1$, and $\Psi_{12}=\Psi_{21}=\frac{1}{4}\left[\sigma {\left(M_{k}(:,1)+M_{k}(:,2)\right)}^{2}-\sigma {\left(M_{k}(:,1)-M_{k}(:,2)\right)}^{2}\right]$.Step 3.Compute the matrix **E** whose columns are the eigenvectors of Ψ, and $\Psi =E\Lambda E^{T}$, where $\Lambda =\mathrm{diag}(\lambda_{1},\lambda_{2})$, and $\lambda_{i}$ are the eigenvalues.Step 4.Let G=DE and $Z_{k}(j,:)={\left(G^{-1}p_{k,j}\right)}^{T}$, and $v={\left[\mu (Z_{k}(:,1)),\mu (Z_{k}(:,2))\right]}^{T}$, and define $\Sigma_{k}\leftarrow c_{1}\Sigma_{k}{}^{\prime }$, and $\mu_{k}\leftarrow c_{2}\mu_{k}{}^{\prime }$, where $\Sigma_{k}{}^{\prime }=G\Gamma G^{T}$, $\mu_{k}{}^{\prime }=Gv$ and $\Gamma =\mathrm{diag}(\sigma {\left(Z_{k}(:,1)\right)}^{2},\sigma {\left(Z_{k}(:,2)\right)}^{2})$,Step 5.Calculate the square of MD $d_{k,j}^{2}$ based on Equation (11) with a threshold of $d_{k0}^{2}=\chi_{2}^{2}(\alpha )$.Step 6.If $d_{s_{k,j}}>d_{k0}$, $p_{k,j}$ is an outlier. Thus, the unreliable probability for $s_{j}$ is 1/m; otherwise, it is 0.

In Algorithm 3, med(·) represents the median value, $\chi_{p}^{2}(\alpha )$ is the α-quantile of the chi-squared distribution with p degrees of freedom, diag(·) is the diagonal matrix, and σ(·) and μ(·) denote the univariate standard deviation and average value, respectively. *c*_1_ and *c*_2_ are a constant. $\Sigma_{k}{}^{\prime }$ and $\mu_{k}{}^{\prime }$ are the estimations of $\Sigma_{k}$ and $\mu_{k}$.

Steps 1–4 in Algorithm 3 provide a method to obtain the positive-definite and approximately equal-variant covariance matrix $\Sigma_{k}$ for high-dimensional scatter datasets with much shorter computing times [19]. The first step in Algorithm 3 makes the position vector scale-equivariant for different dimensions. Then, the GK estimator is used to calculate the covariance matrix Ψ in step 2. However, Ψ is symmetric but not necessarily positive semidefinite, it cannot satisfy the requirement of positive definiteness of $\Sigma_{k}$ [20]. Considering the fact that, the eigenvalues of a covariance matrix can be seen as the variances along the directions of respective eigenvectors, the eigenvalue decomposition is performed to find eigenvalues and eigenvectors in step 3. A modification is then made in step 4 by using the positive robust variances calculated by Equation (12) to replace the eigenvalues, which may be negative [21], to obtain the positive diagonal covariance matrix Γ. Then, Γ is used to estimate the positive-definite covariance matrix $\Sigma_{k}{}^{\prime }$ instead of Λ. It has been proven in [22] that there exist constant *c*_1_ and *c*_2_, such that the true $\Sigma_{k}$ and $\mu_{k}$ can be approximated by the estimations $\Sigma_{k}{}^{\prime }$ and $\mu_{k}{}^{\prime }$, that is $\Sigma_{k}\leftarrow c_{1}\Sigma_{k}{}^{\prime }$, and $\mu_{k}\leftarrow c_{2}\mu_{k}{}^{\prime }$. For the classical fast minimum covariance determinant (FASTMCD) method [23], $c_{1}$ is defined as follows:

(14) $$c_{1}=\frac{med(d_{s_{k,m+1}},\ldots ,d_{s_{k,N}})}{\chi_{2}^{2}(0.5)},$$

and $c_{2}=1$. Once $\mu_{k}{}^{\prime }$ and $\Sigma_{k}$ are obtained in step 4, the MD for every position vector can be calculated according to Equation (11), which can be rewritten as follows:

(15) $$d_{k,j}^{2}(\mu_{k},\Sigma_{k})=c_{1}^{-1}{\left(p_{k,j}-\mu_{k}\right)}^{T}{\left(\Sigma_{k}{}^{\prime }\right)}^{-1}(p_{k,j}-\mu_{k}).$$

Thus, the outliers are identified by comparing the squared MDs with the defined threshold $d_{k0}^{2}$ obtained in step 5. The choice of the threshold is based on the fact that, when the position matrix $P_{k}∼N(\mu_{k},\Sigma_{k})$, the squared MD $d_{k,j}^{2}$ is distributed as a $\chi^{2}$ random variance with 2 degrees of freedom [22].

To reduce the false-alarm probability, we set an unreliable probability to every node in step 6. If $p_{k,j}$ is detected as outliers, then the unreliable probability of $s_{j}$ is set to be $q_{k,j}=1/m$; otherwise, $q_{k,j}=0$. After *m* position matrices are evaluated, the unreliable probability for every node in $S_{rep}$ can be obtained by $q_{j}=\sum_{k=1}^{m}q_{k,j}$. Thus, the unreliable probabilities for the nodes from $S_{rep}$ have been determined. To identify the unreliable nodes in $S_{ref}$, the detection method is repeated with different reference nodes, which are selected from the set of $S_{rep}$ that have been identified as reliable.

### 3.3. WLS Based on Reliable Probability and Distance

When the unreliable probabilities for all nodes are determined, the WLS method with reliable probability $q_{i}$ and distance ${\hat{r}}_{i}$ from the initial position to node $s_{i}$, *i* = 1, …, *N* is applied to perform localization and can be formulated as follows:

(16) $$\hat{p}={(A^{T}WA)}^{-1}A^{T}WB,$$

where

(17) $$W=\mathrm{diag}(w_{1}/{\hat{r}}_{i},\ldots ,w_{N}/{\hat{r}}_{N}).$$

$w_{i}=1-q_{i}$. The procedure for the proposed localization method is given by Algorithm 4.

**Algorithm 4.** The procedure of the proposed method based on DIG and MD: DIG_MD.(1)Perform the DIG method to determine $\Omega_{ref}=\left\{s_{1},s_{2},\ldots ,s_{m}\right\}$ (m≥3) and $\Omega_{rep}=\left\{s_{m+1},s_{m+2},\ldots ,s_{N}\right\}$, and then calculate *m* position matrices $P_{k}$, k=1,…,m.(2)Identify all the outliers in the matrices $P_{k}$ k=1,…,m based on Algorithm 2.(3)Calculate the unreliable probabilities for nodes in $\Omega_{rep}$.(4)Estimate the source position based on Equation (16).(5)Reselect the nodes with high low unreliable probability $q_{i}<0.5$ from $\Omega_{rep}$ with the new initial position obtained from step 5.(6)Repeat steps 1–4 to estimate the source position as the final localization results.

## 4. Simulations

In this section, we compare the performance of the proposed method, DIG_WD, with that of the PLE, the WLS-based distance method denoted as WLS (i.e., the reliable probabilities for all nodes are 1), and the EM-based method [10] through a series of computer simulations.

We assume that *N* nodes are placed uniformly in an *L* × *L* m^2^ test area with a resolution of $\Delta_{x}$ and $\Delta_{y}$ along the horizontal and vertical directions, respectively. Each node is equipped with a microphone array to estimate the AOA of the target, and 1000 Monte Carlo simulations are conducted for every case based on the parameters L=250, $\Delta_{x}=\Delta_{y}=50$, and α=0.95. Next, *u* randomly selected nodes are assumed to be subject to large noise or interference, and their standard deviation of the estimated bearing error is set to be $\sigma_{2}$; moreover, those of the remaining “reliable” nodes are set to be $\sigma_{1}$, $\sigma_{2}≻\sigma_{1}$. The initial positions for WLS, EM, and DIG_MD are obtained by the PLE.

For comparison purposes, we also apply the detection method, Algorithm 3, to identify the outliers from all IPs calculated by every two bearing lines. Instead of calculating the mean of IPs as the source position, the WLS estimator based on reliable probabilities and distance is also used to determine the position. When *t* (t≤n−1) the IPs can be obtained on the bearing line extending from $s_{i}$, *i* = 1, 2, …, *n*; the unreliable probabilities of $s_{i}$ is q/t if *q* IPs included in the *t* points are identified as outliers from all IPs. Next, the WLS based on Equation (16) is used to find the source position; this method is defined as the IP_WLS method. Furthermore, the center of all IPs after excluding all detected outliers is defined as CIP.

### 4.1. The RMSEs for Different $\sigma_{1}$ and $\sigma_{2}$

**Figure 4:**
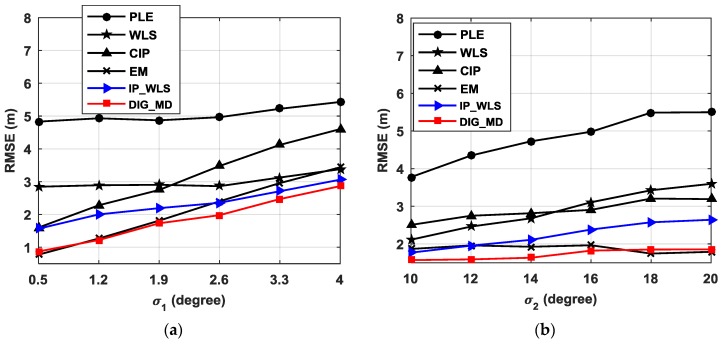
The RMSEs of the pseudolinear estimator (PLE), weighted least squares (WLS), center of all intersected points (CIP), expectation maximization (EM), IP_WLS, and division and greedy replacement–Mahalanobis distance (DIG_MD) methods with (**a**) different $\sigma_{1}$ when $\sigma_{2}=15{}^{\circ}$, (**b**) different $\sigma_{2}$ when $\sigma_{1}=2{}^{\circ}$. (u=6, m=4 ).

The localization performances of various approaches are influenced by the standard deviation of the estimated bearings error. For the source located at $p={\left[73.3,62.3\right]}^{T}$, the RMSEs of different algorithms with different $\sigma_{1}$ are plotted as shown in Figure 4a when $\sigma_{2}=15{}^{\circ}$, u=6 and m=4.

It can be seen that the existence of unreliable bearings can severely deteriorate the localization performance of the PLE, especially when $\sigma_{1}$ is small. When $\sigma_{1}=0.5{}^{\circ}$, the RMSE of the estimated errors for the PLE is as large as 4.83 m. Compared with WLS, the CIP method shows lower RMSEs only when $\sigma_{1}<2{}^{\circ}$, and IP_WLS always outperforms WLS for all values of $\sigma_{1}$, because it is easier to detect the outliers when the data are contaminated severely. This phenomenon also illustrates the importance of detecting outliers. From Figure 4a, we can also observe that IP_WLS shows better performance than CIP, illustrating the superiority of WLS over simple CIP. The EM exhibits a somewhat similar performance to that of DIG_WD when $\sigma_{1}$ is small; however, it shows a greater advantage as $\sigma_{1}$ increases.

The simulation is then conducted when $\sigma_{1}=2{}^{\circ}$ and $\sigma_{2}$ ranges from 10° to 20°, considering the fact that the background noise usually does not change greatly during a short period for certain applications. The results in Figure 4b indicate that the DIG_MD can significantly improve the localization performance compared with the conventional PLE and WLS. CIP can outperform WLS only when the difference between $\sigma_{1}$ and $\sigma_{2}$ is large, and it always has higher RMSEs than those of the IP_WLS method. EM performs slightly better than DIG_MD when $\sigma_{2}$ is significantly larger than $\sigma_{1}$. In contrast, the DIG_MD clearly outperforms EM when $\sigma_{2}$ is less than 16.

From Figure 4a,b, we can see that both IP_WLS and DIG_MD can improve the localization accuracy compared with the PLE and WLS. However, DIG_MD shows better performance than IP_WL. This is because IP_WLS is based on the outlier detection results of IP. These IPs are sensitive to the difference of two AOAs. When the source and two nodes are close to located at a line, the IP will be easily identified as outliers, and thus, false alarm probability will be increased. On the other hand, any of the two AOA errors will have an influence on the IP. When one IP is detected as an outlier, then two nodes will be allocated unreliable probabilities. As a result, the false alarm also exists if only one of them is reliable, especially when the node is close to the source. All these problems can be solved by the proposed DIG method.

### 4.2. The Influence of the Number of Reference Nodes

**Figure 5:**
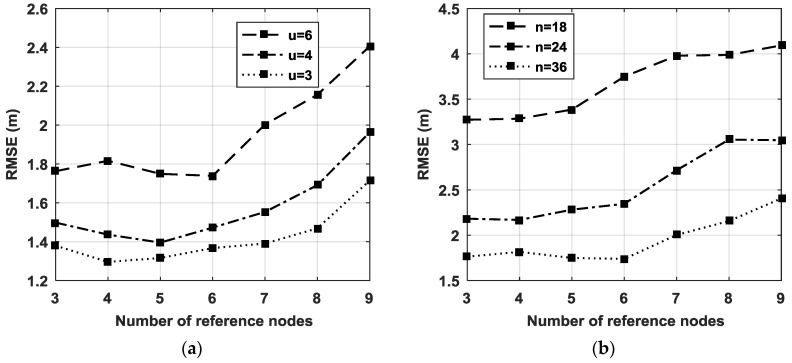
(**a**) The RMSEs of DIG_MD with the number of reference nodes when different unreliable nodes are present and N=36; (**b**) the RMSEs of DIG_MD with the number of reference nodes when different nodes are used and u=6.

To discuss the effect of the number of reference nodes on the localization performance, the RMSEs for different scale of reference nodes are plotted in Figure 5 when $\sigma_{1}=2{}^{\circ}$ and $\sigma_{2}=15{}^{\circ}$.

We can see that more reference nodes should be used when the number of unreliable nodes increases, and the number of reference nodes should be no more than [*n*/6] ([x] is the nearest integer to **x**). Otherwise, the performance of DIG_WD will deteriorate seriously. As illustrated in Section 3, *m* position sets with (*N* − *m*) elements in each set can be obtained using the DIG_MD method. When the number of reference nodes increases, the position sets also increase, whereas the number of estimated locations decreases. Only if there are enough positions to be evaluated should more position sets be used to increase the reliability of detection. To guarantee enough positions in each set to detect outliers, (*N* − *m*) should be significantly greater than *m*. From the simulation results, it can be seen that the reference node number is preferred to be within the range from three to [*N*/6].

### 4.3. The Localization Performance for Different Numbers of Unreliable Nodes

**Figure 6:**
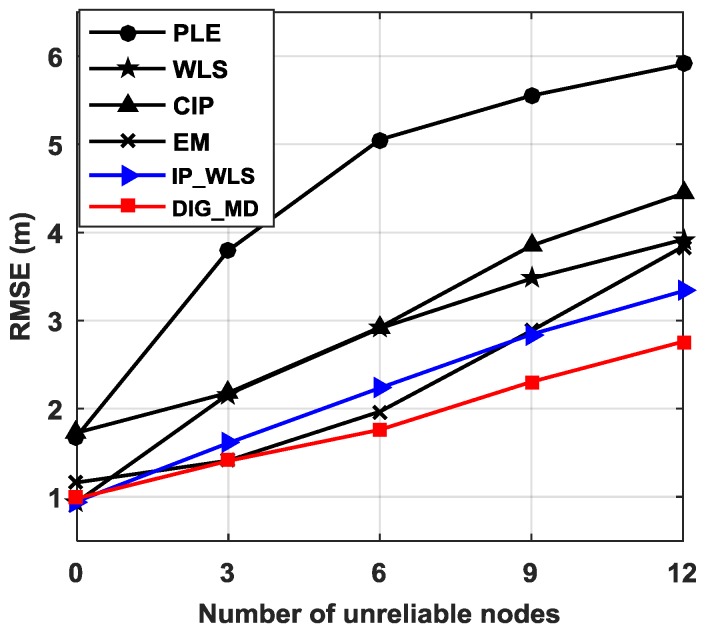
RMSE vs. the number of unreliable nodes when $\sigma_{1}=2{}^{\circ}$ and $\sigma_{2}=15{}^{\circ}$ and m=4.

Figure 6 further shows the localization performance for different numbers of unreliable nodes. It can be seen that the RMSEs of all the methods increase as the number of unreliable nodes increases. Compared with the PLE, CIP can improve the localization performance when unreliable nodes are present; however, it exhibits slightly higher RMSE than PLE when there is no outlier. EM has the highest RMSE among EM, WLS, IP_WLS, and DIG_MD; however, it performs better than WLS when the number of unreliable nodes increases. The IP_WLS and DIG_MD methods can inhibit the effect of unreliable bearing measurements for all cases. The superiority of the DIG_MD method over other methods increases as the number of unreliable nodes increases.

**Figure 7:**
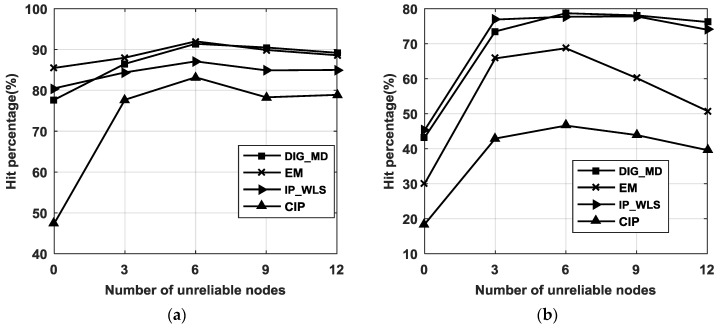
(**a**) Hit percentages of the DIG_MD, EM, IP_WLS, and CIP methods compared with the PLE when $\sigma_{1}=2{}^{\circ}$ and $\sigma_{2}=15{}^{\circ}$, and m=4; (**b**) hit percentages of the DIG_MD, EM, IP_WLS, and CIP methods compared with WLS when $\sigma_{1}=2{}^{\circ}$ and $\sigma_{2}=15{}^{\circ}$, and m=4.

To investigate the robustness of the proposed method, the hit percentage of DIG_MD (when the errors of the evaluated methods are less than WLS or the PLE) is shown in Figure 7. The figure shows that the CIP method has the lowest hit percentages compared with both the PLE and WLS. EM has higher hit percentages than IP_WLS compared with the PLE, while the latter can improve localization accuracy with greater probability than EM compared with WLS. In contrast, the hit percentages of DIG_MD retain its superiority compared with both the PLE and WLS.

### 4.4. The Localization Performance for Different Numbers of Nodes and for Different Source Positions

**Figure 8:**
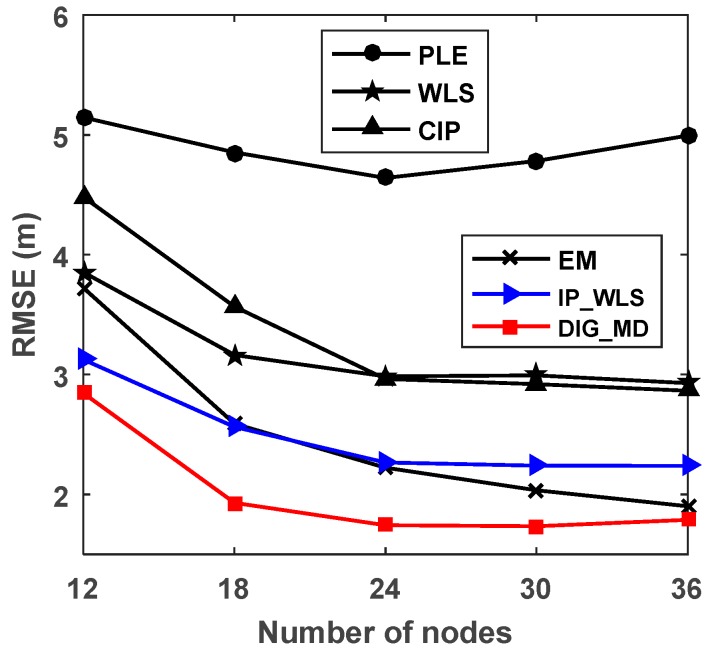
RMSE of PLE, WLS, CIP, EM, IP_WLS and DIG_MD vs. the number of nodes when $\sigma_{1}=2{}^{\circ}$, $\sigma_{2}=15{}^{\circ}$.

As the number of nodes usually has a great influence on the localization performance, we plot the relationship between RMSE and the number of nodes in Figure 8. For fairness, the number of unreliable nodes is *N*/6. The number of reference nodes is four. Figure 8 shows that EM has a higher RMSE than IP_WLS and DIG_MD methods when the number of nodes is 12. However, the IP_WLS method shows worse performance than EM as the number of nodes increases. The proposed method, DIG_MD, always has the best localization accuracy for the different cases.

**Figure 9:**
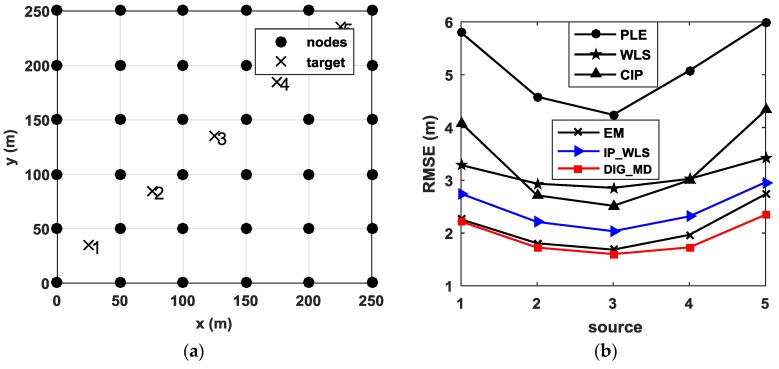
(**a**) Five target positions in a 36-node distributed network; (**b**) RMSE for different target positions when m=4, u=5, $\sigma_{1}=2{}^{\circ}$ and $\sigma_{2}=15{}^{\circ}$.

To study the efficiency of the proposed method for different source positions, Figure 9b shows the localization performance for five different source positions when six unreliable measurements are present. It is clear that the proposed method can improve the localization performance significantly for all source positions.

## 5. Outdoor Experiment Results and Analysis

**Figure 10:**
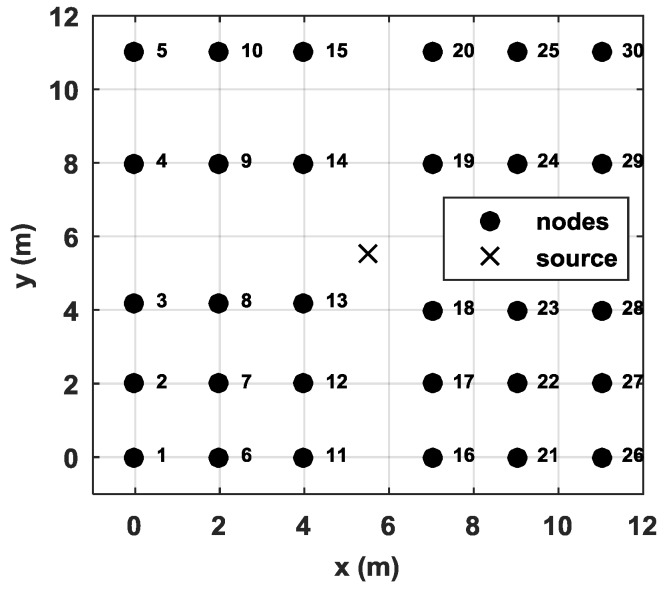
Node placement for the 35-node network.

**Figure 11:**
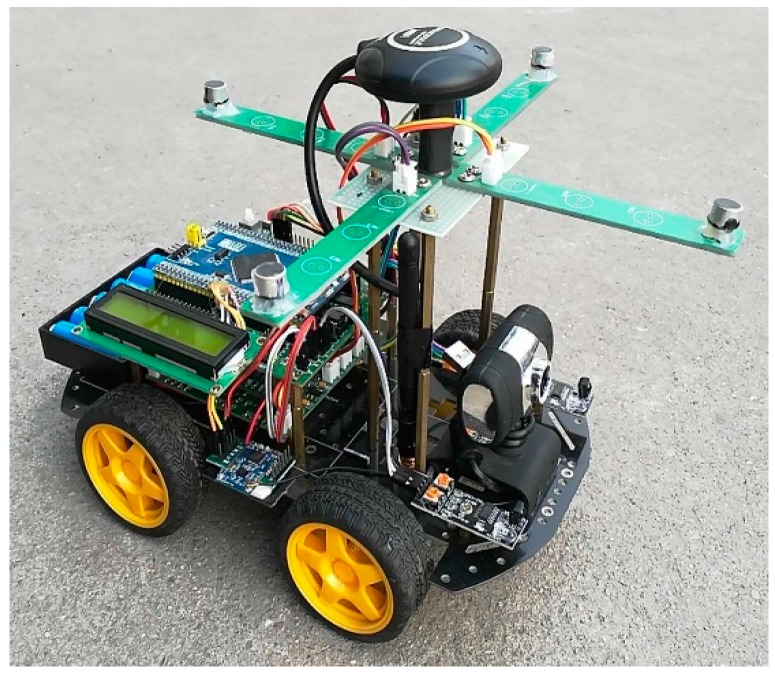
The four-element cross-microphone array.

In this section, we describe the verification of our proposed method using a 30-node network for acoustic source localization. All nodes were placed in an $11\times 11\;m^{2}$ square field, as shown in Figure 10. Each node is an autonomous vehicle equipped with a four-element cross microphone array, as shown in Figure 11. The microphone array is arranged into two orthogonal pairs 20 cm apart. Each pair of microphones estimates an AOA using the generalized cross correlation with phase transform (GCC-PHAT) [24] method. The final AOA is then obtained by the fusion of two AOAs obtained by the two pairs of microphones. The vertical distance from ground to microphone is also 20 cm. During the test, all nodes transmitted the estimated angles to the base station following a predefined collision-avoidance communication protocol. The localization tests were repeated 40 times. The acoustic source was a car engine noise generated by a loudspeaker orientated upward. Without loss of generality, we placed the speaker at the center of the test field (i.e., $x={\left[5.5m,5.5m\right]}^{T}$).

The experiment is conducted in an outdoor environment, with noise always present. However, the signal-noise-ratio (SNR) for each node is different as the distances from source to nodes are different. The range is from 5 to 15 dB. During the experiment, unreliable AOAs may be introduced by the following:

- Multipath signal: because the distance between the microphone array and the ground is only 20 cm, unreliable AOAs may be introduced by a multipath signal.
- Interferences: the movements of people and cars during the experiment are also causes of unreliable measurements.
- The low SNR: because of the possible nonstationary background, the SNR of the received signal of each node may vary in a large range, possibly resulting in unreliable measurements.

**Figure 12:**
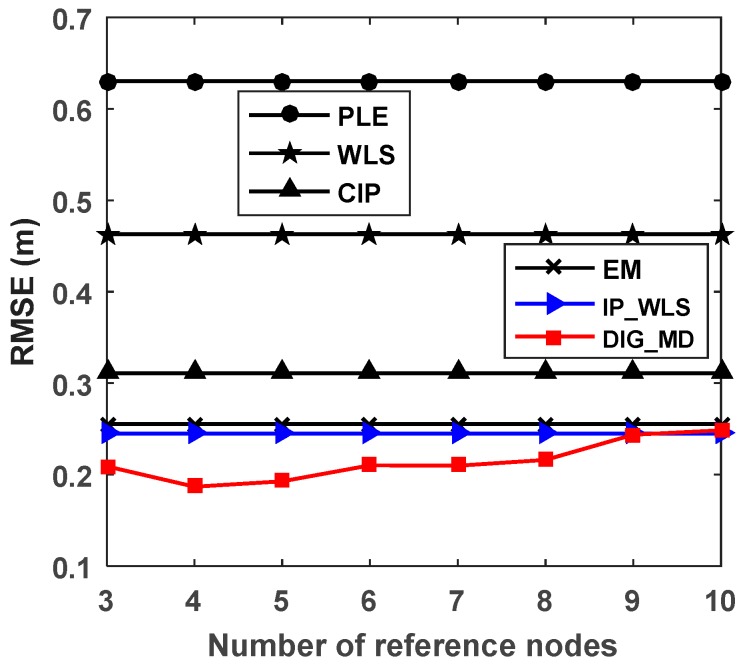
RMSEs for different numbers of reference nodes.

**Figure 13:**
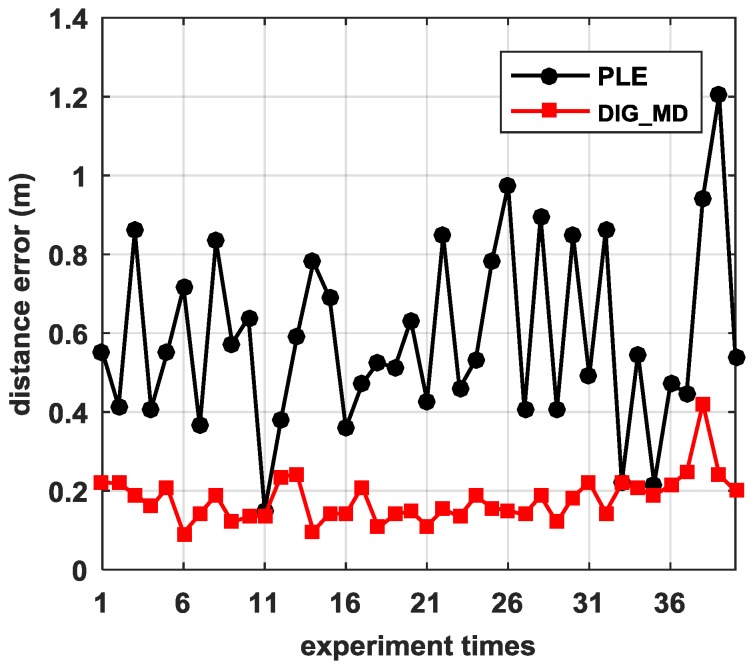
The localization error for 30 repetition estimation results.

**Figure 14:**
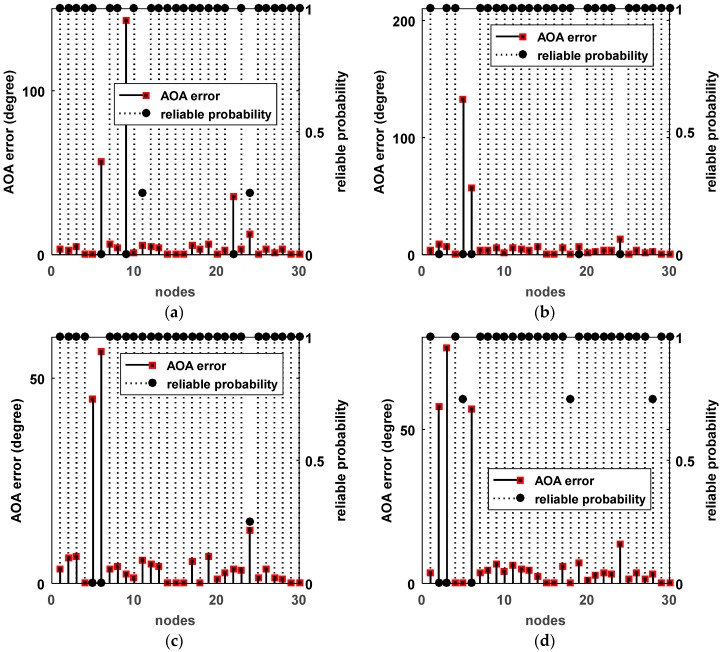
Estimated AOA errors and unreliable sensor detection results. (**a**) The 11th experiment; (**b**) the 33rd experiment; (**c**) the 10th experiment; (**d**) the 32nd experiment.

To verify the influence of the number of reference nodes on the localization accuracy, we plotted the RMSEs of different numbers of reference nodes, as shown in Figure 12. We can see that the proposed method DIG_MD clearly outperforms other compared methods when the number of reference nodes is fewer than six. As the number exceeds six, the RMSEs of DIG_MD increase gradually. When more than nine reference nodes are used, the DIG_MD method yields similar localization performance to the IP_WLS. To show the localization results more clearly, we further compared the localization results of DIG_MD with the PLE for the 40 experiments with m=4, as shown in Figure 13. The results show that while most of the large error peaks of PLE were substantially degraded, there were a few cases in which the DIG_MD method performed slightly better than the PLE (e.g., in the 11th and 33rd runs). To investigate the underlying reason, we plotted the unreliable sensor node detection results for the two cases, as shown in Figure 14a,b. For comparison purposes, the estimated AOA values and the detection results for the 10th and 32nd experimental runs for which the proposed method significantly improves the localization performance are plotted in Figure 14c,d, respectively.

As nodes, $s_{11},s_{24}$, are misjudged as unreliable for the 11th run, the localization error of DIG_MD is only slightly better than that of the PLE, even though the unreliable nodes $s_{6},s_{9},s_{22}$ can be detected; a similar situation can also be found in the 33rd experiment. In contrast, the unreliable nodes in the 10th run can be detected correctly. For the 32nd run, the localization error can be significantly decreased while $s_{18}$ and $s_{28}$ are detected with a very low false-alarm probability.

**Figure 15:**
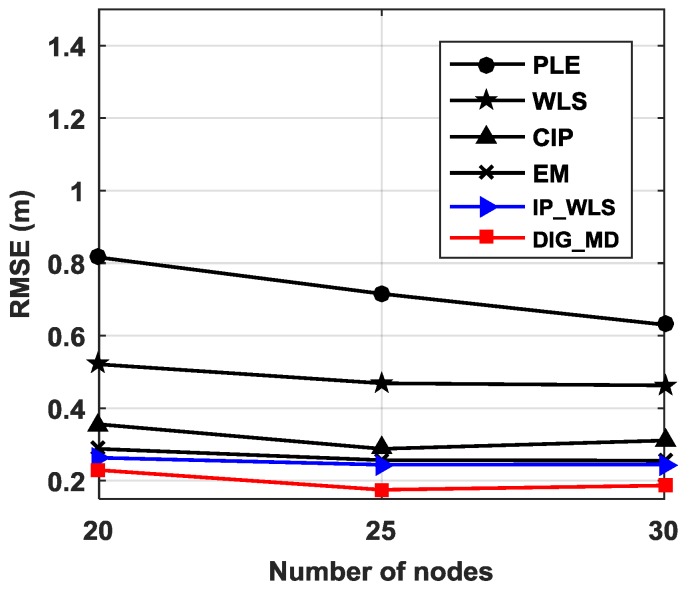
RMSEs for different numbers of nodes.

To verify the localization performance under different numbers of nodes in a node network, we only use $s_{1}∼s_{k}$, k=20,25,30 to perform localization when four reference nodes are used. Note that when different numbers of nodes are used, the source location is no longer located at the center of all nodes. The simulation results shown in Figure 15 reveal that DIG_MD has the best localization performance for all the cases considered.

## 6. Conclusions

The localization performance of conventional AOA-based method, the PLE, is prone to be deteriorated when unreliable measurements are present. In this paper, we propose an unreliable node detection method based on the characteristics of the estimated positions of different node combinations. In the proposed approach, the DIG method is used to acquire different position sets, and the MD based on robust location and covariance matrix estimator is used to identify the outliers from the estimated target position sets. The proposed method does not require any prior information about the target and is easy to implement. Both simulation and outdoor experiment results show that DIG_MD is efficient and robust against the influence of unreliable measurements and can significantly improve the localization accuracy when the measurements are contaminated.

## Figures and Tables

**Table 1:** RMSE for different numbers of nodes.

Unreliable Node	*s*_1_	*s*_2_	*s*_3_	*s*_4_
RMSE (m): *N* = 4	1.2201	0.3168	0.3146	0.8060
RMSE (m): *N* = 3	0.2155	0.1642	0.1695	0.1780
